# A comprehensive review on apoptotic miRNAs and antagomiRs in breast cancer: Enhancing radio sensitization

**DOI:** 10.1016/j.gendis.2025.102024

**Published:** 2026-04-22

**Authors:** Maryam Najafi-Amiri, Mina Pourhabib-Mamaghani, Seyed Hamid Aghaee-Bakhtiari, Mohammad-Taghi Bahreyni-Toossi, Hosein Azimian

**Affiliations:** a Department of Medical Physics, Faculty of Medicine, Mashhad University of Medical Sciences, Mashhad 9919191778, Iran; b Department of Medical Biotechnology and Nanotechnology, Faculty of Medicine, Mashhad University of Medical Sciences, Mashhad 9919191778, Iran; c Bioinformatics Research Center, Basic Sciences Research Institute, Mashhad University of Medical Sciences, Mashhad 9919191778, Iran; d Medical Physics Research Center, Basic Sciences Research Institute, Mashhad University of Medical Sciences, Mashhad 9919191778, Iran

**Keywords:** Apoptosis, Breast cancer, microRNA, Radioresistance, Radiosensitivity

## Abstract

Breast cancer (BC) is the most fatal cancer in women worldwide, and in spite of progresses in cancer treatment methods, its therapy has remained as a primary health issue. Inadequate apoptosis level is one of the main reasons of radiotherapy and chemotherapy resistance in lots of cancers including BC. Besides, gene therapy is one of new methods which has been proposed and developed in recent decades for various diseases diagnosis and treatment containing cancer. Since changes in miRs level which are one of different types of RNA molecules have been proved in assorted diseases, they are utilized as a gene therapy agent in order to make influence in diverse signaling pathways which are effective in unappealing biological alteration. In lots of studies, miRs’ role in apoptosis pathway in combination with radiotherapy, chemotherapy or solely have been investigated. Many miRNAs and anti-miRNAs have been demonstrated as radiosensitizers/radioresistance agents and chemosensitisers/chemoresistance agents through apoptosis cell death pathways, and numerous miRs have been introduced as apoptotic regulators and they have a good potential to be radiosensitizers and their role in these pathways must be investigated. In the current review, we have tried to investigate and summarize radiosensitizer and radioresistant miRs and anti-microRNAs (anti-miRs) which have been utilized in BC.

## Introduction

In 2020, an estimated 19.3 million cancer cases were reported worldwide. Among these, female breast cancer (BC) accounts for 11.7% of the new cases (about 2.3 million people), making it the most common type of cancer. This was followed closely by lung cancer, which accounted for 11.4% of all cancer cases. Moreover, BC mortality, with about 6.9% of death rate from all cancers, ranked fifth; both its incidence and mortality are increasing annually.^1^^,^^2^

Treatment strategies for BC are prescribed based on the tumor's grade, stage, subtype and molecular features, and may include surgery, radiation therapy, chemotherapy, targeted therapy, endocrine therapy, immunotherapy and so on. Stage 0 BC is typically treated using surgery and radiotherapy with or without endocrine therapy. Nonmetastatic stage 1 and 2 are often treated with immunotherapy or endocrine therapy, prechemotherapy or chemotherapy (especially for TNBC, which lacks all three receptors), surgery and radiotherapy. Stage 4 BC is considered incurable, and its treatment is primarily palliative.^3^ Among the common methods for BC treatment, radiation therapy is one of the main techniques which is utilized alone or in combination with other methods, definitive and adjuvant treatment. Also, radiotherapy can be applied to early-stage, locally advanced and metastatic BC using diverse techniques. The applied techniques consist of conventional whole breast radiotherapy, hypo-fractionated whole breast radiotherapy, partial breast radiotherapy, accelerated partial breast radiotherapy and so forth.^4^^,^^5^

One of the crucial responses to ionizing irradiation and chemotherapy agents in many cancers is apoptotic cell death. Inactivation of apoptotic or increasement of anti-apoptotic signaling pathways can lead to escape of cancerous cells from apoptosis and cause an uncontrolled repopulation. So, low apoptosis rate can lead to treatment resistance in radiotherapy and apoptosis-dependent chemotherapy. Therefore, enhancing apoptosis is a critical strategy for increasing radiosensitivity and chemosensitivity in cancer treatment, including BC.^6^, ^7^, ^8^, ^9^ Studies have demonstrated a correlation between the slope of the survival curve after irradiation and apoptosis resistance in various cancerous cell lines. In cell lines resistant to apoptotic cell death, the survival curve exhibits a moderate slope and a smooth initial shoulder. The lower resistance to apoptosis, the higher slope of the survival curve following irradiation, and the less resistance to radiotherapy.^10^ Consequently, increasing the level of apoptosis is one of the main goals of many pharmacological and therapeutic methods and strategies in cancer treatment including BC.

Also, another one of new methods for many diseases diagnosis and treatment is gene therapy which has been brought up in recent years and consists of using genes or their mRNA regulators including siRNAs, miRNAs, antimiRs and so on.^11^, ^12^, ^13^, ^14^ These nucleic acids can be effective in numerous disorders including cancer and various pathways such as metastasis, apoptosis, proliferation etc.

**Figure 1:**
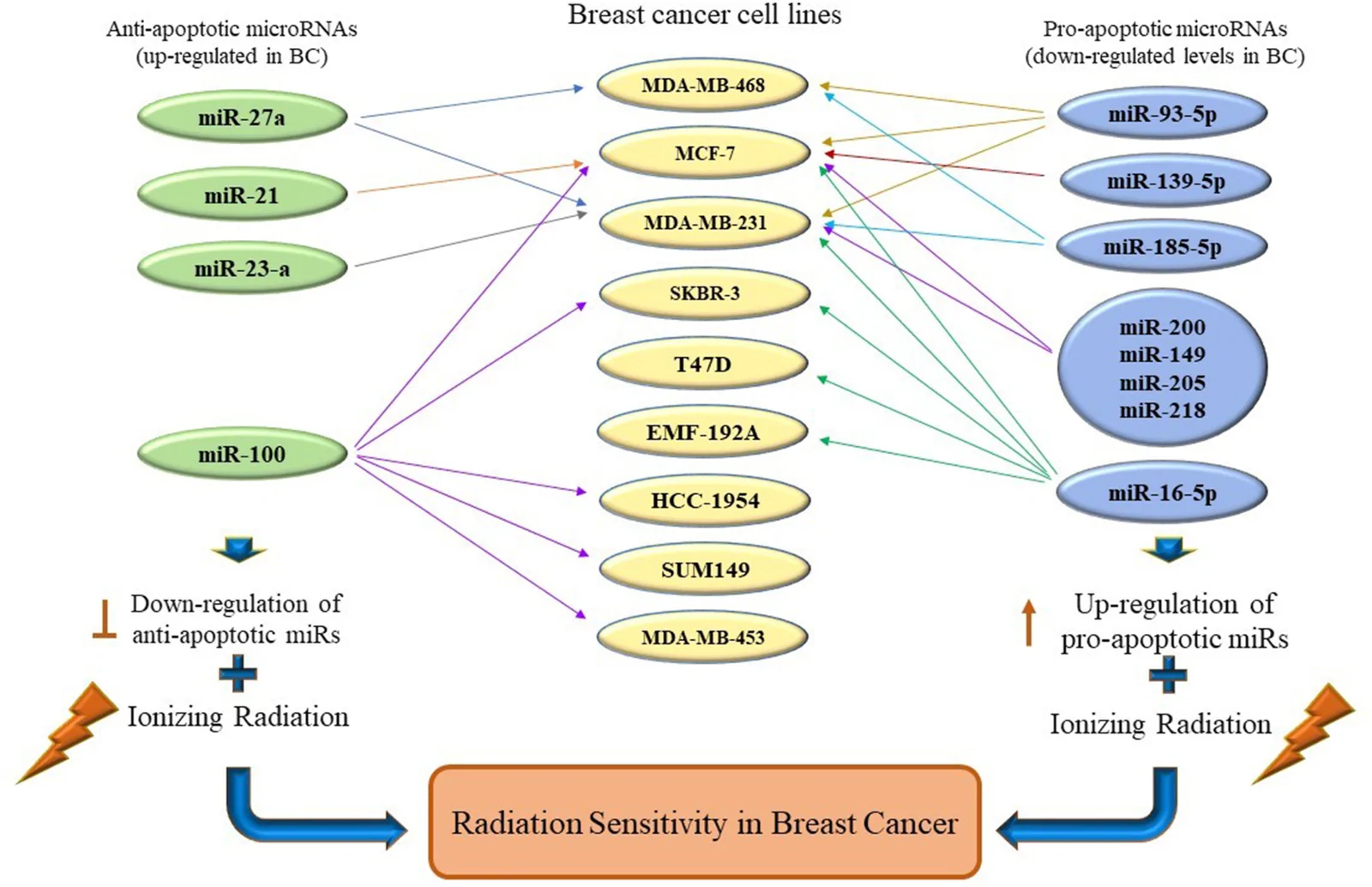
Apoptotic radiosensitizer miRs and antimiRs which have been utilized in various breast cancer cell lines.

**Figure 2:**
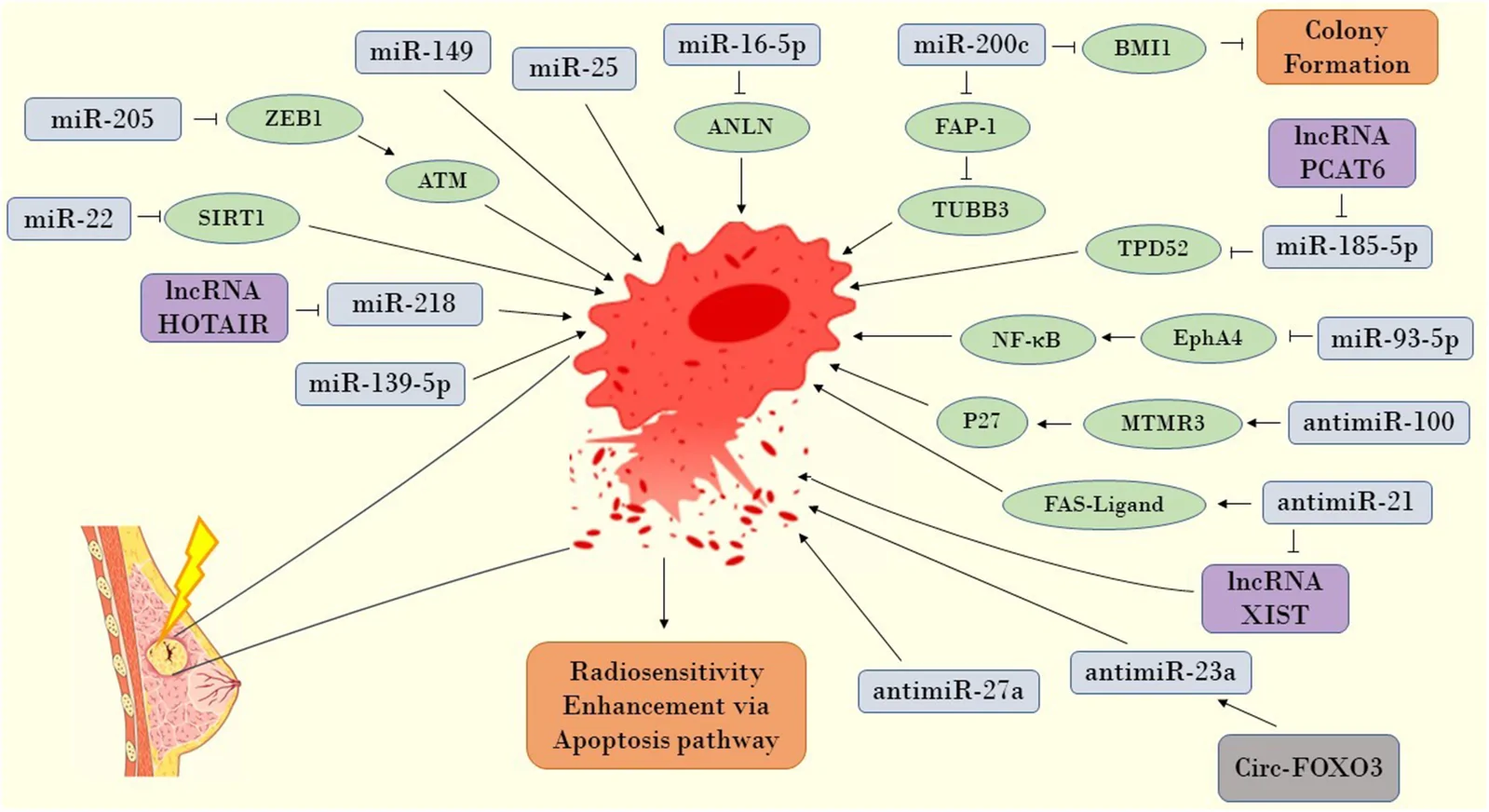
Apoptotic radiosensitizer miRNAs and anti-miRNAs and related pathways in breast cancer.

Considering the above, as it demonstrated briefly in Figures 1 and 2, this review aims to summarize and discuss the role of apoptotic cell death in breast cancer radio-resistance. Next, we will discuss the influence of nucleic acids on apoptosis. Ultimately, the specific involvement of apoptotic miRNAs and antagomiRs which participate in BC apoptotic cell death in conjunction with ionizing irradiation will be investigated, focusing on enhancing BC radio-sensitivity.

## Apoptosis in cancer treatment—a spatial look at breast cancer

There are three principal forms of programmed cell death (PCD) forms: apoptotic cell death, autophagy and programmed necrosis.^15^ The concept of apoptosis, also known as programmed cell death or cell suicide, was first introduced in 1972 by Kerr et al.^16^ The main morphological characteristics include nuclear fragmentation and the formation of apoptotic vesicles.^17^ Two main pathways for apoptosis have been found out, containing the cell intrinsic or mitochondrial signaling pathway and the cell extrinsic signaling pathway.^18^ The cell intrinsic apoptosis pathway largely depends on the Bcl-2 (B cell lymphoma 2) protein family and the release of cytochrome C from mitochondria, occurring after specific intracellular alterations such as DNA damages.^19^, ^20^, ^21^ In contrast, the cell extrinsic apoptosis pathway is initiated via the activation of cell death receptors located on the cell membrane surface by pro-apoptotic ligands.^22^

Despite the fact that apoptotic cell death is an essential pathway for embryonic development and maintaining homeostasis in adults in most of normal tissues, there are certain conditions -such as spontaneous or external agents-induced mutations-cells lose their ability to undergo apoptosis. This loss causes an increment in cell proliferation and the induction of various diseases, including neurodegenerative diseases and cancer initiation. So, in many types of cancer cells, overexpression of anti-apoptotic molecules or down-regulation of pro-apoptotic molecules occurs, allowing cancerous cells to resist numerous apoptosis pathways.^23^, ^24^, ^25^ For example, alterations have been demonstrated in APC6, P5 and h*MSH3* in colorectal cancer,^26^ TP53, NF1, IDH1, BCL2, BCL2L2, BOK in glioblastoma,^27^^,^^28^ CASP8, BID and BARF1 in gastric cancer,^29^, ^30^, ^31^ Fau, Bcl-G, Bcl2 and VDAC1 in prostate cancer,^32^^,^^33^ CRC6 in oral squamous cell carcinoma,^34^ CARM1 in liver cancer^35^ and so on. Since apoptosis is the primary death signaling pathway following irradiation and some of chemotherapy agents, cancerous cell lines with these deregulations show radio-resistance, chemo-resistance and tumor recurrence, resulting in low survival rates for patients.

In various types of cancers, reduction in tumor cell proliferation and increased median survival have been reported with the combination of radiotherapy or chemotherapy and anti-apoptosis inhibitors or pro-apoptotic agents. For instant, a modified doxorubicin nanoparticle (NP), used alongside radiation therapy, significantly inhibited the proliferation of non-small lung cancer tumor cells and enhanced apoptosis compared to radiation therapy or chemotherapy alone.^36^ In glioblastoma, small molecule XIAP inhibitors induced apoptosis and enhanced radio-sensitization in cancerous cells.^37^ Similarly, in anaplastic thyroid cancer (ATC), Livin-knockdown led to increased irradiation-induced apoptosis and inhibited tumor cells invasion.^38^ In addition, nasopharyngeal carcinoma exhibited increased apoptotic cell death after treatment with a combination of radiotherapy and cetuximab.^39^ Thus, various anti-apoptotic agents can be utilized alongside radiotherapy and/or chemotherapy to enhance apoptosis rate and radio-sensitization in different types of cancers.

The apoptosis pathway is also demonstrated to be altered in BC. Survival curves in human, mouse and other species’ BC cell lines have shown resistance to apoptosis.^40^^,^^41^ Additionally, it has been shown that there is a clear relationship between apoptosis resistance and tumor grade, aggressiveness, metastasis and local recurrence in BC.^42^^,^^43^ Similar to other cancers, BC can lose its balance between apoptotic cell death and proliferation through various altered pathways. For instance, TP53 mutations have been confirmed in different subtypes of BC.^44^^,^^45^ Furthermore, Several anti-apoptotic proteins, such as BAG3 and HSP70 have been shown to be down-regulated in BC, particularly in TNBC.^46^ Enas A. et al reported that apoptotic and antiapoptotic factors change in the plasma and tumor cells of patients with different stages of BC, with bcl-2 and sFas being up-regulated in BC tumor cells.^47^ These alterations contribute to BC resistance to chemotherapy, radiotherapy, endocrine therapy and other treatments.^46^^,^^48^ However, it has been reported that with increased hypoxia-induced apoptosis in the breast cancer tumor microenvironment, increased fusion of tumor cells and mesenchymal stem cells occurs. This leads to increased metastasis probability.^49^

Taken together, strategies based on targeted enhancement of the level of programmed cell death (apoptosis) in breast cancer without harming healthy tissues appear to increase treatment efficacy, the likelihood of tumor control, and patient survival.

## Nucleic acids’ role in apoptosis

Nucleic acids were first proposed as treatment approximately 50 years ago. Nowadays, nucleic acids are widely being explored and applied in the diagnosis and treatment of many human diseases, including cancer. Various disorders, such as leukemia, lymphomas, myeloma, sarcoma, adrenoleukodystrophy, wiskott-aldrich syndrome, adenosine deaminase deficiency, chronic granulomatous disease and epidermolysis bullosa, are candidates for gene therapy.^50^^,^^51^ Additionally, several types of ribonucleic acids are utilized in gene therapy, including mRNAs, miRNAs, antimiRs, siRNAs, LncRNAs and so on.^52^

Different mechanisms can be targeted and involved in gene therapy for specific diseases, and one of the substantial pathways for cancer gene therapy is the induction of inflammation-free apoptotic cell death.^53^ This objective can be achieved by inhibiting anti-apoptotic factors or up-regulating pro-apoptotic factors. This goal can be accomplished via utilizing various pathways. For example, previous studies demonstrated that Bcl-x antisense oligonucleotides and hsa-miR-7-5p usage in order to suppress anti-apoptotic genes cause apoptosis enhancement in the PC-3 prostate cancer cell line.^54^^,^^55^ It's shown that P53-mRNA NPs, acting as tumor suppressors, improve outcomes in hepatocellular carcinoma and non–small cell lung cancer cells. This NP sensitizes theses cancerous cells to mTOR inhibitors.^56^ STAT3 activation has been implicated in BC progression and apoptosis resistance. The siRNA pRNAi-Stat3 affected the expression of BcL-xL, Fas, Fas-L, Casp-3, Casp-8 and Casp-9, leading to apoptosis induction in MDA-MB-231 BC cells.^57^ In glioma, lncRNA TUG1 was identified as a pro-apoptotic agent via influencing the expression of Casp-3, Casp-9 and Bcl-2 in intrinsic apoptosis pathways.^58^ Down-regulation of lncRNA NEAT1 led to growth inhibition and facilitated apoptosis in colorectal cancer, positioning it as a diagnostic biomarker and therapeutic agent for this disease.^59^ In HOS and MG-63 osteosarcoma cells, hsa_circ_0001564, a type of circRNA, was reported to be overexpressed and function as a pro-apoptotic miR-29c-3p sponge. Knockdown of has_circ_0001564 mediated osteosarcoma tumorigenicity, causing cell cycle arrest, apoptosis induction and inhibited proliferation.^60^ miR-506-3p levels are down-regulated in both ovarian cancer tissues and cell lines. Its overexpression caused inhibited proliferation and promoted apoptotic cell death through inactivation of the SIRT1/AKT/FOXO3a pathway.^61^ miR-125b is up-regulated in prostate cancer, and its inhibition via anti-miR-125b induced apoptotic cell death through alterations in p14ARF and Mdm2 expression levels.^62^ Many other studies have investigated the effects of various nucleic acids on apoptosis and proliferation in different types of cancers. These studies have reported numerous sequences as promising novel diagnostic and therapeutic agents.

## MiRNAs and antimiRs’ role in apoptosis and radio-sensitivity

MiRNAs are small noncoding RNAs, typically 19 to 25 nucleotides in length, which play a substantial role in post-transcriptional gene regulation and restrain their target mRNA function. As a result, miRNAs can influence many pathways and lead to alterations in their related proteins. Moreover, it has been reported the level of miRNAs changes in many diseases compared to healthy cells. These features make it possible to utilize miRNAs in the diagnosis and treatment of many diseases, including cancer.^63^^,^^64^ It has been proved that cancer can result from an imbalance between cell division and apoptotic cell death. So, one of the major roles of miRNAs in cancer induction, drug resistance, radio-resistance, and low overall survival rate occurs through the apoptotic signaling pathway.^65^, ^66^, ^67^ Furthermore, as mentioned earlier, the most important signaling pathway of cell death due to irradiation and some chemotherapy drugs is apoptotic cell death. So, resistance to apoptosis signaling pathways causes radio-resistance and chemo-resistance.

AntimiR or antisense oligonucleotide, also referred to as antagomiR, is a type of ribonucleic acids (RNA), which is involved in cellular signaling pathways. AntimiR oligonucleotides can block miRNAs by binding to them, effectively ‘sponging’ them up. Thereby, antimiRs prevent miRNAs from exerting their function and influencing mRNAs. Consequently, these nucleic acid sequences participate in various cellular signaling pathways, including apoptosis. So, their regulation alters radio-resistance and chemo-resistance levels in different cancers.^68^^,^^69^

Numerous studies have investigated the role of miRNAs and antimiRs in modulating apoptosis signaling pathways. For instance, miR-181a has been shown to effectively regulate PRKCD expression. Up-regulation of miR-181a has been observed in radio-resistant SiHa and Me180 squamous cervical carcinoma cells in both *in vitro* and *in vivo* models. Furthermore, the use of miR-181a inhibitor increased the radio-sensitivity of cancerous cells.^70^ miR-124, a STAT3 activator, was found to be down-regulated in non-small-cell lung carcinoma tissue and A549 cell line. Also, enhancing miR-124 levels increased radio-sensitivity and apoptosis.^71^ In contrast, miR-208a, inducible by radiation, promotes proliferation and reduces apoptotic cell death in A549 non-small cell lung cancer cells through targeting p21.^72^ Additionally, miR-99a was found to be highly expressed in sensitive A549 cell lines. Also, its induction led to increased apoptotic cell death and radio-sensitivity via targeting the mTOR pathway.^73^ Therefore, it can be concluded that in A549 non-small cell lung cancer cells miR-208a and miR-99a may act as radio-resistant and radio-sensitive apoptosis regulators agents, respectively. In prostate cancer LNCaP cell line, the overexpression of miR-29b-3p increased radio-sensitivity and mitochondrial apoptosis rates. The changes caused decreasing proliferation, cell viability and colony formation ability.^74^

In glioma cells, miR-196b has been reported to exhibit enhanced oncogenic activity and a substantial role in the PI3K/AKT pathway. Therefore, anti-miR-196b combined with irradiation and chemotherapy, increased apoptosis and reduced colony formation ability in U87 and U251 cell lines.^75^ Similarly, silencing miR-21 using anti-miR-21 induced G2/M checkpoint inhibition, apoptosis and radiation sensitivity in T47D and MDA-MB-361 BC cell lines.^76^

These findings, along with numerous other studies, demonstrate the efficacy of miRNAs in modulating the apoptosis signaling pathway for cancer treatment, both *in vitro* and *in vivo*.

## MiRNAs and apoptosis induction in breast cancer cells after radiation

Radiation therapy plays an essential role in the management of BC treatment, particularly following breast-conserving surgery. The primary goal of radiotherapy is to eliminate any remaining cancer cells, lower the likelihood of recurrence, and improve overall survival rates. Latest investigations have demonstrated that numerous miRNAs can play a role in promoting apoptosis in BC cells following radiation exposure. In the subsequent section, we will discuss the studies that examined miRNAs and antimiRs in conjunction with radiation in detail. The key findings from related investigations are concisely summarized in Table 1 and Table 2.

**Table 1:** Summery of radiation sensitive and pro-apoptotic miRNAs in BC.

miR	*In vitro*/*vivo* condition	Pathway(s)	Ref.
miR-16-5p	• MCF-7 • MCF-10A • MDA-MB-231 • T47D • EMF-192A • SKBR-3	miR-16-5p reduces breast cancer cell growth and movement by targeting the anillin (ANLN) gene, leading to increased cell death, especially when combined with radiation therapy.	77
miR-200	• MDA-MB-231 • MCF-7	miR-200c controls cell death, targets FAP-1, lowers TUBB3 for chemo effectiveness and increased sensitivity to IR, leading to elevated apoptosis levels in breast cancer cells.	78
miR-149	• MCF-7 • MDA-MB-231	miR-149 blocks basal BC cell migration and invasion by targeting SP1, FOXM1, and ZBTB2. Irradiation activates miR-25 and miR-149 in breast cancer, boosts apoptosis, and reduces resistance.	79,80
miR-185-5p	• MCF-10A • MDA-MB-468 • MDA-MB-231	Knocking down PCAT6 or TPD52, or overexpressing miR-185-5p, increased radiosensitivity in TNBC cells by reducing cell proliferation and increasing apoptosis after radiation.	81
miR-93-5p	• MCF-7 • MDA-MB-231 • MDA-MB-468	Elevated miR-93 is tied to aggressive breast cancer, poor prognosis. Combined IR and miR-93-5p treatment increased apoptosis more in MDA-MB-231 cells, increasing radiosensitivity synergistically.	82 83
miR-139-5p	• MCF-7	miR-139-5p affects radiotherapy response by regulating DNA repair genes and ROS defense pathways. Overexpression inhibits repair and lowers free radical removal, increasing oxidative stress. miR-139-5p treatment boosts breast cancer cell sensitivity to radiation, enhancing apoptosis and sensitizing BC cells to radiation.	84
miR-22	• MDA—MB-231 • MCF-7	miR-22 enhances cancer treatment by targeting SIRT1 and roles as radiosensitizer in BC cells by increasing apoptosis.	85
miR-205	• MDA-MB-231 • MCF-7	Radiation decreases miR-205 via ataxia telangiectasia mutated (ATM), zinc finger E-box binding homeobox 1 (ZEB1) and ubiquitin-conjugating enzyme (Ubc13), that leading to DNA repair inhibition and cell apoptosis.	86
miR-218	• MDA-MB-231 • MCF-7	Inducing apoptosis in breast cancer cells, miR-218 with radiation and HOTAIR knockdown increases DNA damage and cell cycle arrest, impacting cell survival.	87

**Table 2:** Radiation resistant and anti-apoptotic miRNAs in BC. These miRNAs can be inhibited utilizing their antimiRs.

miR	Cell line(s)	Inhibit apoptosis pathway	Ref.
mir-21	• MCF-7	miR-21 inhibits Fas ligand (FasL) in BC cells, supporting immune evasion. Targeting miR-21 may enhance FasL-mediated apoptosis, and aiding immunotherapy against tumors. Also, inhibition of mir-21 Affects the expression of LNCRNA X-Inactive and led to apoptosis.	88,89
miR-27a	• MDA-MB-231 • MDA-MB-468	miR-27a is an oncogene in cancers like BC, regulating processes like proliferation and invasion by directly targeting components of the AKT signaling pathway. In TNBC, upregulated miR-27a promotes tumorigenesis by aiding cell growth and survival.	90
miR-23a	• MDA-MB-231	Overexpressing miR-23a after RT suppressed apoptosis.	91

## miR-16-5p

miR-16-5p is a miRNA which plays a significant role in the progression of numerous malignancies, including neuroblastoma, osteosarcoma, hepatocellular carcinoma (HCC), cervical cancer, BC, brain tumors, gastrointestinal cancers, lung cancer, and bladder cancer.^92^ Interestingly, elevated miR-16-5p levels have been associated with improved prognosis in BC patients, correlating with higher radiosensitivity and survival rates. This suggests that it may function as a biomarker for radio-sensitivity and the overall effectiveness of treatment. Mechanistically, miR-16-5p impacts cell cycle dynamics, particularly by triggering G2/M phase arrest. This arrest can improve the responsiveness of cancer cells to irradiation, thus increasing the likelihood of apoptosis following treatment.^93^ So, considering its function as a tumor suppressor, strategies to increase miR-16-5p levels in BC cells may improve the efficacy of radiation therapy. This could be achieved through the application of miRNA mimics or other techniques to restore its expression in tumor tissues in which it is diminished.^92^

Evidence indicates that miR-16-5p targets the ANLN gene, which plays a crucial role in cell proliferation and migration processes. By inhibiting ANLN, miR-16-5p effectively diminishes the proliferation of BC cells and facilitates apoptosis, particularly in conjunction with radiation therapy.^77^

## miR-200c

Previous investigations have found out miR-200c suppresses the tumorigenicity of human breast cancer stem cells (BCSCs) by inhibiting cell growth and inducing differentiation.^94^ It also inhibits colony formation in breast cancerous cells by modulating BMI1 expression. Additionally, miR-200c regulates apoptosis by targeting FAP-1 and restores sensitivity to chemotherapy by reducing TUBB3 levels.^95^ Taken together, miR-200c inhibits migration, invasion, stem cell growth, tumorigenicity, and chemo-resistance. A research conducted on two BC cell lines, MCF-7 (high levels of miR-200c expression) and MDA-MB-231 (low levels of miR-200c expression), demonstrated that miR-200 can enhance radio-sensitivity through apoptotic signaling pathways.^78^ Additionally, the results of this research demonstrated that overexpression of miR-200c in cancerous cells increased sensitivity to ionizing irradiation, leading to elevated apoptosis in BC cells. Treatment with miR-200c significantly increased apoptotic cell death level compared to the miR-NC (negative control), both with and without irradiation. miR-200c alone and in combination with radiation resulted in a more than 3 times higher percentage of apoptotic cells at 24 h post-irradiation (8 Gy) in both MCF-7 and MDA-MB-231 cells.

## miR-25 and miR-149

E. Abdollahi et al investigated the effects of 4 and 8 Gy radiation doses on apoptosis induction in two BC cell lines (MCF-7 and MDA-MB-231). The results have shown that miR-25 and miR-149 can be triggered by irradiation in BC to enhance the apoptosis rate and decrease BC radio-resistance in both MCF-7 and MDA-MB-231 cell lines. However more research is needed to investigate the pathway of these miRNAs’ effects.^80^

## miR-185-5p

Recent investigations have identified the miR-185-5p/TPD52 axis as a crucial pathway in TNBC, influencing radio-sensitivity and tumor progression. Notably, the down-regulation of miR-185-5p and the up-regulation of PCAT6 and TPD52 in TNBC tissues are linked to aggressive tumor characteristics, resulting in poor clinical outcomes such as lymph node metastasis and advanced disease stages. Exposure to various doses of x-rays (2, 4, 6 and 8 Gy) along with the overexpression of miR-185-5p demonstrated the knockdown of TPD52 in TNBC cells (MDA-MB-468 and MDA-MB-231) and breast epithelial cells (MCF-10A). These alterations led to enhancement of the radio-sensitivity, reduction of cell proliferation and significant increment of apoptosis in MDA-MB-468 and MDA-MB-231 cells following radiation therapy.^81^

## miR-93-5p

miR-93-5p is associated with tumor progression and poor overall survival in TNBC patients. Likewise, various studies have explored the role of miR-93-5p in BC and its influence on radiation sensitivity, suggesting potential implications for treatment strategies. One investigation, by Chi Pan et al, studied the effect of miR-93-5p on the radiation sensitivity of BC cell lines (MDA-MB-468, MCF-7, and MDA-MB-231) following 2 Gy irradiation. They found that combining radiation with miR-93-5p treatment led to significantly higher apoptosis than either treatment alone, especially in the MDA-MB-231 (*p* < 0.005) and MCF-7 (*p* < 0.001) cell lines. This suggests a synergistic effect, potentially enhancing radiation sensitivity.^82^ Also, miR-93-5p accompanied by irradiation caused to tumor suppression and apoptosis enhancement in MDA-MB-231 TNBC cell line both *in vitro* and *in vivo* via miR-93-5p/EphA4/NF-κB signaling pathway.^83^

## miR-139-5p

miR-139-5p plays a crucial role in modulating the response to radiotherapy by regulating DNA repair genes and reactive oxygen species (ROS) defense pathways. Overexpression of miR-139-5p inhibits DNA repair mechanisms and reduces the scavenging of free radical, leading to increased oxidative stress in irradiated BC cells. Moreover, treatment using miR-139-5p mimic demonstrated that enhancing its expression increases the sensitivity of BC cells to radiation by accumulating unrepaired DNA damage, resulting in enhancement of apoptosis (*p* < 0.05). In summary, up-regulation of miR-139-5p is anticipated to enhance treatment outcomes BC patients undergoing radiotherapy, and its overexpression sensitizes MCF7 cells to irradiation.^84^

## miR-22

Previous investigations have consistently demonstrated that miR-22 in combination with various doses of radiation (2, 4, 6 and 8 Gy), effectively led to apoptosis induction in two prominent human BC cell lines, MDA-MB-231 (*p* < 0.05) and MCF-7 (*p* < 0.05). Notably, miR-22 exerts its anti-tumor effects by targeting SIRT1, thereby enhancing radiation sensitivity of BC cells and suppressing tumorigenic potential.^85^

## miR-205

Irradiation at doses of 2, 4 and 6 Gy reduces the expression level of miR-205 by acting on ATM and ZEB1. miR-205 as a radiosensitizer hinders DNA repair mechanisms and survival fraction (*p* = 0.02), and induces apoptosis in MDA-MB-231 and MCF-7 cell lines.^86^

## miR-218

In addition to the previously mentioned miRNAs, miR-218, in combination with 8 Gy irradiation and HOX antisense intergenic RNA (HOTAIR) knockdown, can also induce apoptosis in MCF-7 and cell line. HOTAIR expression increases after irradiation, and its knockdown decreases cell survival, increases apoptosis, and enhances DNA damage and cell cycle arrest in response to radiation. The radiation sensitizing effects of HOTAIR are linked to the up-regulation of miR-218. Thus, the use of miR-218 inhibitor + shHOTAIR led to a significant reduction in apoptosis compared to the shHOTAIR group (*p* < 0.05).^87^ Also, the results of the study by Wischmann et al show that targeting miR-218 expression leads to increased radiosensitivity and reduced cell growth in MDA-MB-231 line (*p* < 0.05) through the EGFR pathway, which is one of the signaling pathways effective in the apoptosis process.^96^

## anti-miR-21

miR-21 has been identified to be up-regulated and causes resistance to radiotherapy or chemotherapy treatment in many types of cancers such as breast cancer, ovarian cancer, glioblastoma, pancreatic cancer, renal carcinoma and so on.^97^, ^98^, ^99^ It has been confirmed that miR-21 inhibits FAS ligand mediated immune responses in MCF-7 cell line and leads to apoptosis reduction.^88^ Also, anti-miR-21 has reduced lncRNA-XIST expression and increased apoptosis in MCF-7 BC cell line from 7.52% to 31.5%.^89^ High-dose irradiation induced exosomal miR-21 in BC has been discovered to increase proliferation in 4T1 BC cell line.^100^ So, anti-miR-21 has a potential to increase radio-sensitivity and improve BC treatment efficacy.

## anti-miR-27a

miR-27a has increased proliferation in MDA-MB-231 and MDA-MB-468 TNBC both *in vivo* and *in vitro* and anti-miR-27a has caused growth inhibition in these cell lines. In addition, miR-27a mimic decreased caspase-3/7 and BAX expression, as well as increased Bcl-2 in both cell lines, indicating a reduction in apoptosis.^90^ Thus, anti-miR-27a can be a candidate for radio-sensitivity enhancement.

## anti-miR-23a

miR-23a and FOXO3 expression has been found to be enhanced and down-regulated in BC patients, respectively. Also, it has been showed that circ-FOXO3 can predict cellular radiosensitivity and miR-23a overexpression has leaded to apoptosis inhibition in radioresistant MDA-MB-231 BC cell line following various doses of gamma irradiation (2, 4 and 8 Gy).^91^

Following the introduction of miRNAs that enhance (Table 1) or inhibit (Table 2) apoptosis in BC cells in response to radiation, and act as radio-sensitizers, Table 3 lists some of the additional miRNAs which induce apoptosis in BC cells, but have not yet been studied in combination with radiation. These miRNAs may also be effective for radiation sensitization:

**Table 3:** Some of the apoptotic miRNAs which have not been investigated in combination with radiation therapy.

miR	Cell line(s)	Pathway	Ref.
miR-100	• SK-BR-3 • MCF7 • MDA-MB-453 • T47D • HCC1954 • SUM149 • MDA-MB-231 • HS578T • BT549 SUM159	miR-100 is upregulated in SK-BR-3 BC, MDA-MB-231, HS578T, BT549 and SUM159 cells compared to others. Targeting miR-100 with anti-miRNA oligonucleotides increases apoptosis and cell cycle arrest by activating the p27 protein, enhancing cancer cell response to chemotherapy for improved efficacy.	101 102
miR-497	• MCF-7	miR-497 triggers cell death by targeting Bcl-w, slowing cell growth and increasing apoptosis in BC cells, suggesting its potential as a tumor suppressor and role as radiosensitizers.	103
miR-3188	• HCC1954 • MDA-MB-231 • SK-BR-3 • BT474	miR-3188 enhances IR sensitivity by affecting the mTORC2/AKT signaling pathway by altering the expression of Rictor, which could be a promising therapeutic strategy for the future treatment of breast cancer	104
miR-372	• MCF-7	miR-372 suppresses cell growth and triggers cell death in MCF-7 breast cancer cells by targeting E2F1 directly.	105
miR-34b-5c (miR-34c)	• MDA-MB-231	miR-34c demonstrated powerful anti-cancer effects, inhibiting cell growth, migration, and promoting apoptosis.	106

## Conclusion

In numerous studies, the effectiveness of miRNAs has been investigated in the initiation, progression, metastasis, treatment resistance, recurrence, and their related signaling pathways in different types of BC. Lots of miRNAs have been identified as tumor suppressors, oncogenes, or bi-functional agents in BC, affecting apoptosis primary related pathways. Anti-apoptotic and pro-apoptotic miRNAs can influence cell proliferation and modify radio-sensitivity in various BC cell lines, both *in vitro* and *in vivo*.

Pro-apoptotic miRNAs bind to mRNA transcripts of anti-apoptotic genes, increasing the rate of apoptotic cell death. These pathways can exhibit synergistic effects with radiotherapy in BC. miR-16-5p, which is produced by MIR16-1 gene, has been identified to restrain NF-κB, AKT3, ANLN and VEGFA. It acts as a tumor suppressor and pro-apoptotic microRNA in BC.^92^ Also, lncRNA MIR497HG/miR-16-5p axis is an effective signaling pathway in BC progression.^107^ As a result, it can suppress breast cancerous cell cycle dynamics, migration, proliferation, and act as radiation sensitizer and a pro-apoptotic agent.^77^^,^^93^ Furthermore, miR-200c level is downregulated in BC patients. It has been shown that its expression negatively impacts tumorigenicity, stemness, cell growth, epithelial–mesenchymal transition and colony formation in various BC cell lines, including breast cancer stem cells. This is achieved by modulating the expression of FAP-1, BMI1, KRAS, SOX2, SNAIL, TWIST1, KLF4 and TUBB3. Moreover, up-regulation of miR-200c can increase apoptotic cell death in BC cells, particularly when combined with irradiation.^78^^,^^95^^,^^108^ Similar pro-apoptotic effects in BC have also been observed with miR-93-5p,^82^^,^^83^ miR-25 and miR-149.^80^ Though, contrary to what has been demonstrated about miR-93-5p role in radiosensitivity, high miR-93-5p level has been reported to be linked to low overall survival rate, aggressive tumor characteristics and unfavorable outcomes in BC. Also, the overexpression of miR-93-5p leads to chemotherapy resistance through the Wnt/β-catenin signaling pathway.^82^^,^^83^ So, its role in radiosensitivity requires further investigation in different BC cell lines. miR-185-5p, which is a target of lncRNA SNORD3A,^109^ has been identified as a radio and chemo sensitizer in BC. This miRNA promotes apoptosis through the TPD52, PCAT6 and UMPS pathways.^81^^,^^109^ miR-139-5p has been shown to impact ROS and DNA repair pathways in BC, and can also function as an apoptotic radio-sensitizer.^84^ Additionally, miR-3188, miR-22, miR-205 and miR-218 act as radio-sensitizers by influencing the mTORC2/AKT, SIRT1, ATM/ZEB1 and HOTAIR pathways.^104^ miR-21, which has been extensively studied, is up-regulated and exhibits oncogenic properties in various cancers, including radio-resistant BC. Specifically, it inhibits DNA damage repair and G_2_ checkpoint arrest, and its antisense oligonucleotide has been found to enhance apoptotic cell death levels following irradiation and chemotherapy in BC.^76^^,^^110^^,^^111^

Other miRNAs have been reported to be effective in apoptosis pathways in BC cell lines, either alone or in combination with chemotherapy. These miRNAs could also be explored in conjunction with irradiation as potential radio-sensitizers. miR-100, which has been described as a bi-functional apoptotic miRNA in different cancers, has been found at high levels in malignant BC cell lines. However, some studies have reported that miR-100 has reduced in murine 4T1 metastatic BC cell line compared with nonmetastatic 67NR.^112^ Researches have shown that anti-miR-100 can enhance apoptotic cell death along with chemotherapy.^101^ However, its impact on irradiation and radio-sensitization remains an unexplored area of research.

In addition to antagomiRs, long non-coding RNAs (lncRNAs) are functional RNA molecules, which can regulate level of miRs, as well. Many lncRNAs have been reported to be effective in apoptosis pathways in BC.^113^, ^114^, ^115^, ^116^ LncRNA HOTAIR and lncRNA CCAT1 increased after irradiation in BC cells. Reduction of these LncRNAs caused a radio-sensitization effect related to miR-218, miR-324-3p and miR-148b, respectively.^87^^,^^117^ LncRNA GAS5 has been shown to be chemo-sensitizer and radio-sensitizer, and its enhancement led to an increase in apoptosis via miR-21 in BC.^118^ This reveals that lncRNAs, as antagomiRs, could be appropriate ribonucleic acids with radio-sensitivity or radio-resistance roles through their effects on miRNAs.

In conclusion, BC is the most prevalent lethal cancer among women worldwide, with its incidence increasing annually. Radiotherapy is one of the primary treatment strategies for BC. However, radiation resistance, caused by resistance to apoptosis, can lead to treatment failure or cancer recurrence. By modulating apoptosis signaling pathways, miRNAs and antagomiRs have emerged as potential tumor suppressors that enhance radio-sensitivity in various cancers, including BC. This review explores the role of miRNAs in apoptosis, with a specific focus on pro-apoptotic and anti-apoptotic miRNAs utilized in conjunction with radiation therapy for BC. Numerous miRNAs have been investigated for their apoptotic effects, either alone or in combination with chemotherapy. These include miR-497,^103^ miR-17/20,^119^ miR-17-5p,^120^ miR-363,^121^ miR-520b,^122^ miR-153-3p,^123^ miR-34a,^110^ among others. However, their potential as radio-sensitizers in radiation therapy has not yet been researched.

Evidences suggest that several miRNAs and antagomiRs act as radiation sensitizers by promoting apoptosis in BC, indicating their potential therapeutic application in combination with ionizing radiation to improve treatment effectiveness.

## CRediT authorship contribution statement

**Maryam Najafi-Amiri:** Writing – review & editing, Writing – original draft, Methodology. **Mina Pourhabib Mamaghani:** Writing – original draft. **Seyed Hamid Aghaee-Bakhtiari:** Writing – review & editing. **Mohammad-Taghi Bahreyni-Toossi:** Writing – review & editing. **Hosein Azimian:** Writing – review & editing, Methodology.

## Funding

This work was supported by the 10.13039/501100004748Mashhad University of Medical Sciences (Iran).

## Conflict of interests

The authors declare they have no conflict of interests.
